# “Liberation treatment” for chronic cerebrospinal venous insufficiency in multiple sclerosis: the truth will set you free

**DOI:** 10.1002/brb3.297

**Published:** 2014-11-21

**Authors:** Georgios Tsivgoulis, Simon Faissner, Konstantinos Voumvourakis, Aristeidis H Katsanos, Nikos Triantafyllou, Nikolaos Grigoriadis, Ralf Gold, Christos Krogias

**Affiliations:** 1 Second Department of Neurology, “Attikon” Hospital, School of Medicine, University of AthensAthens, Greece; 2 International Clinical Research Center, Department of Neurology, St. Anne's University HospitalBrno, Czech Republic; 3 Department of Neurology, University of Tennessee Health Science CenterMemphis, TN; 4 Department of Neurology, St. Josef-Hospital, Ruhr UniversityBochum, Germany; 5 Department of Neurology, School of Medicine, University of IoanninaIoannina, Greece; 6 First Department of Neurology, “Eginition” Hospital, School of Medicine, University of AthensAthens, Greece; 7 Department of Neurology, Laboratory of Experimental Neurology and Neuroimmunology, AHEPA Hospital, Aristotle University of ThessalonikiThessaloniki, Greece

**Keywords:** Chronic cerebro-spinal venous insufficiency, iron, “Liberation” treatment, multiple sclerosis, transcranial sonography, ultrasound, venous angioplasty

## Abstract

**Background:**

Chronic cerebrospinal venous insufficiency (CCSVI) has recently been introduced as a chronic state of impaired cerebral or cervical venous drainage that may be causally implicated in multiple sclerosis (MS) pathogenesis. Moreover, percutaneous transluminal angioplasty of extracranial veins termed “Liberation treatment” has been proposed (based on nonrandomized data) as an alternative therapy for MS.

**Methods:**

A comprehensive literature search was conducted to identify available published, peer-reviewed, clinical studies evaluating (1) the association of CCSVI with MS, (2) the reproducibility of proposed ultrasound criteria for CCSVI detection (3) the safety and efficacy of “Liberation treatment” in open-label and randomized-controlled trial (RCT) settings.

**Results:**

There is substantial heterogeneity between ultrasound case–control studies investigating the association of CCSVI and MS. The majority of independent investigators failed to reproduce the initially reported high prevalence rates of CCSVI in MS. The prevalence of extracranial venous stenoses evaluated by other neuroimaging modalities (contrast or MR venography) is similarly low in MS patients and healthy individuals. One small RCT failed to document any benefit in MS patients with CCSVI receiving “Liberation treatment”, while an exacerbation of disease activity was observed. “Liberation treatment” has been complicated by serious adverse events (SAEs) in open-label studies (e.g., stroke, internal jugular vein thrombosis, stent migration, hydrocephalus).

**Conclusion:**

CCSVI appears to be a poorly reproducible and clinically irrelevant sonographic construct. “Liberation treatment” has no proven efficacy, may exacerbate underlying disease activity and has been complicated with SAEs. “Liberation treatment” should stop being offered to MS patients even in the settings of RCTs.

## Introduction

Multiple sclerosis (MS) is a chronic, primary inflammatory disease of the central nervous system (Gold et al. 2006). In 2009, a new concept for the pathogenesis of MS based on the idea of an impaired cerebrospinal venous drainage was postulated, differing from the established concept of the multifactorial pathogenesis of MS (Zamboni et al. 2009a). This hypothesis was based on specifically developed ultrasound features which were stated to detect cervical or cerebral abnormalities of venous drainage leading to increased intracranial venous pressure, subsequently followed by blood–brain barrier breakdown causing iron deposition in brain parenchyma initiating the development of MS (Zamboni 2006). Moreover, percutaneous transluminal angioplasty of extracranial veins (termed “Liberation treatment”) has been proposed (based on nonrandomized data) as an alternative therapy for MS by the same group of investigators introducing CCSVI hypothesis (Zamboni et al. 2009c). Despite the lack of higher class evidence “Liberation treatment” has gained a considerable amount of attention and emotional involvement by MS patients worldwide (Chafe et al. 2011) and has started to be offered as a potential therapeutic option in MS patients in nonrandomized and uncontrolled studies (Hubbard et al. 2012; Mandato et al. 2012; Ghezzi et al. 2013b).

However, numerous independent investigators failed to detect any association between CCSVI neurosonology criteria and MS in numerous case–control studies, while “Liberation treatment” has been complicated with serious adverse events (SAEs) leading to substantial criticism of “venous hypothesis” of MS pathogenesis (Barkhof and Wattjes 2013; Valdueza et al. 2013).

## Methods

In view of the former considerations, we conducted a comprehensive literature search to identify available published, peer-reviewed, clinical studies evaluating (1) the association of CCSVI with MS using different ultrasound modalities, (2) the reproducibility of proposed ultrasound criteria for CCSVI detection, (3) the safety and efficacy of “Liberation treatment” in open-label and randomized-controlled trial (RCT) settings.

Our literature search through MEDLINE was based on the combination of terms “Chronic cerebro-spinal venous insufficiency”, “multiple sclerosis”, “transcranial sonography”, “iron”, “ultrasound”, “Liberation treatment,” and “venous angioplasty”. Last literature search was conducted on 14 August, 2014. Reference lists of all articles that met the criteria and of relevant review articles were examined to identify studies that may have been missed by the database search. Titles, abstracts and, whenever appropriate, full texts of all identified studies were screened independently by two reviewers in English (GT, CK) and two reviewers in German (SM, CK) journals. Potential disagreements were resolved by consensus of all contributing authors. Duplicate publications and publications in other than the English or German language were excluded from further evaluation.

## Results

### The venous hypothesis of the pathogenesis of MS

The “venous hypothesis” postulates that disturbances of the venous drain from the cervical and/or spinal venous system leads to a congestion and increase in the intracranial venous pressure (Singh and Zamboni 2009). Given his experience in vascular surgery, Dr Zamboni who introduced the “CCSVI hypothesis” saw pathophysiological parallels between a chronic state of cervical or cerebral venous insufficiency and MS (Zamboni 2006). Similar to the chronic venous insufficiency (CVI) in the leg veins, he postulated that cerebral venous stasis may induce endothelial damage leading to blood–brain barrier disruption, which in turn may result in extravasation of erythrocytes (Zamboni 2006). He also hypothesized that disintegrating erythrocytes may be dismounted by macrophages which may cause a local deposition of iron (Ackermann et al. 1988). The focal increased amount of iron may in turn induce a chronic inflammatory reaction with an upregulation of the migration of leukocytes in the subcutaneous matrix via expression of adhesion molecules (ICAM, VCAM) and of selectins (Colleridge-Smith et al. 1988). He underlined that macrophages and T-lymphocytes will play a predominant role in this process, since macrophages have been shown to phagocyte the accumulated iron in subcutaneous tissue and store it intracellularly (Wilkinson et al. 1993; Takase et al. 2004). Moreover, the CCSVI hypothesis underscores that extracellular depositions and iron-loaded macrophages are not only the histopathological feature of CVI but can also be found in MS plaques (Adams et al. 1989) and have been shown to stimulate the immune system (Weilbach et al. 2004). The fact that MS-plaques are located in the perivenous region led to the hypothesis that the pathophysiology of MS may be mediated through a chronic inflammatory reaction whose cause lays in the impaired venous outflow (as well as in CVI).

This hypothesis acquired a high resonance in some patient groups as well as in the media, as it was presented as a comprehensible, potentially curable cause of MS which was easy to understand (Pullman et al. 2013). Moreover, patient organisations and advocacy groups, scientific societies, and health-care authorities started funding CCSVI at an International level (Canada, Italy, United States) under pressure from the media and the blogosphere (Pullman et al. 2013).

### External validation of ultrasound criteria for CCSVI detection

**Figure 1:**
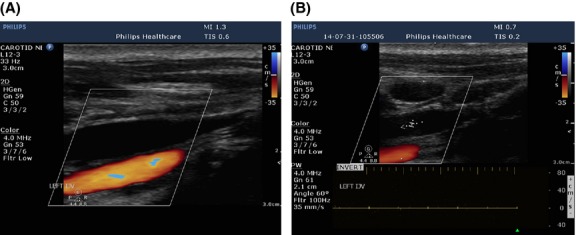
Flow not-Doppler detectable in the Internal Jugular Vein (Criterion IV) in horizontal color-flow image before (A) and after (B) spectral interrogation.

Zamboni et al. reported in their pivotal study that neurosonology had 100% accuracy parameters (sensitivity, specificity, positive predictive value, negative predictive value) to discriminate MS patients from Healthy Controls (HC) using a set of ultrasound criteria developed to detect impaired cervical or venous drainage (Zamboni et al. 2009b). Consequently, they introduced evidence of two positive out of five proposed ultrasound criteria as necessary condition for CCSVI diagnosis (Table 1, Fig.1) (Zamboni et al. 2009a). Moreover, they recommended a detailed neurosonology protocol for CCSVI screening (Nicolaides et al. 2011). However, it should be noted that blinding of sonographers was suboptimal in the majority of studies of Zamboni's group.

**Table 1:** Proposed ultrasound criteria for CCSVI diagnosis (at least two criteria present)

Criterion	Description
I	Reflux constantly present in internal jugular veins (IJV) or vertebral veins (VVs) with the head at 0° (supine position) and +90° (upright position) assessed as flow reversal from its physiologic direction for a duration of >0.88 sec during a short period of apnea following a normal exhalation reflux constantly present in Internal Jugular Vein (IJV) and or Vertebral Vein (VV)
II	Reflux in deep cerebral veins (DCVs) assessed as the presence of flow reversal for a duration of >0.50 sec during normal breathing in at least one of the following three DCVs: basal vein of Rosenthal (BVR), great vein of Galen (GVG), and internal cerebral vein (ICV)
III	High-resolution B-mode evidence of proximal IJV stenosis (defined as local reduction in cross-sectional area > 50% or cross-sectional area <0.3 cm^2^ at the supine position)
IV	Flow not Doppler detectable in the IJVs and/or VVs with the head positioned at 0° (Fig.1) and +90°
V	Reverted postural control of the main cerebral venous outflow assessed as negative difference of the cross-sectional area (CSA) in the IJVs measured in the supine position subtracted from the cross-sectional area in the IJVs measured in the upright position

A series of studies conducted by independent investigators failed to reproduce the findings reported by Zamboni and coworkers in their pivotal studies. In a first ad-hoc investigation of a small unselected group of MS patients in Bochum, the prevalence of CCSVI was similar in MS patients (20%) and HC (10%) (Krogias et al. 2010). In a larger investigation conducted in Berlin, CCSVI criteria were not met in a single individual out of 56 investigated patients (Doepp et al. 2010). In a North-American study using sonographers trained by Zamboni, a higher prevalence of CCSVI was documented in MS patients (63%) than in HC (26%) (Zivadinov et al. 2011). Moreover, a post hoc analysis indicated that CCSVI was independently associated with a more progressive MS course. The association of CCSVI and MS was also reproduced by a Jordanian (Al-Omari and Rousan 2010) and a Polish (Zaniewski et al. 2013) group of investigators that were also offering “Liberation treatment” in their MS patients.

In contrast to the previous observations, an Italian study, investigating only MS patients with clinically isolated syndrome, reported a normal ultrasound investigation in 84% of study population (Baracchini et al. 2011). Moreover, CCSVI could not be detected in additional series of MS patients investigated in Greece (Tsivgoulis et al. 2011) and Germany (Frankfurt/Giessen) (Mayer et al. 2011). In addition, the largest to date, methodologically robust (using both local and central blinded readers) ultrasound case–control study involving 1874 subjects from 35 Italian centers reported a similar (very low) prevalence of CCSVI in MS (3%) and HC (2%) (Comi et al. 2013). Interestingly, the poor interrater and intrarater agreement in CCSVI ultrasound criteria reported both by Italian (Comi et al. 2013) and Greek (Tsivgoulis et al. 2011) investigators underscore the lack of reproducibility of the proposed neurosonology protocol (Table 2). More specifically, the positive agreement between central and local readers in the Italian study was disappointedly low (18%) (Comi et al. 2013). Finally, in an assessor-blinded, case–control, multicentre Canadian study using doppler ultrasound equipment identical to that used in the pivotal study by Zamboni et al. (2009a) and experienced sonographers trained in the center of Zamboni in Ferrara, the prevalence of CCSVI was similar in MS (44%) and HC (45%) (Traboulsee et al. 2014).

**Table 2:** Inter- and intrarater agreement of ultrasound criteria for CCSVI diagnosis

Study	Number of patients	Zamboni's Group	Findings
Menegetti et al. 2010	36 (*12 MS, 12 HC, 12 OND*)	Yes	1 Interrater reliability between trained and not trained sonographers in Zamboni's center: *κ *= 0.47
			2 Interrater reliability between trained sonographers: *κ *= 0.80
			3 Intra-rater reliability in trained sonographers: *κ *= 0.93
Tsivgoulis et al. 2011	15 (*8 MS, 7HC*)	No	1 Interrater reliability regarding criterion I, III & IV: *κ *= 0.82–1.00
			2 Interrater reliability regarding Criterion II & IV: *κ *= 0.14–0.48
Zivadinov et al. 2011	36 (*11 MS, 14 HC, 3 OND*)	Yes	1 Interrater reliability: not available
			2 Intra-rater reliability: *κ *= 0.75
Comi et al. 2013	1767 (*1165 MS, 376 HC, 226 OND*)	No	1 Interrater reliability between local and central readers: *κ *= 0.13
			Negative agreement: 92% (90–93%)
			Positive agreement: 18% (13–22%)

MS, Multiple sclerosis; HC, Healthy Controls; OND, Other Neurological Disorders.

In view of the discrepant results between different investigators, recent meta-analyses (Laupacis et al. 2011; Krogias et al. 2013; Zwischenberger et al. 2013; Tsivgoulis et al. 2014) have suggested an independent association between an ultrasound-based diagnosis of CCSVI and MS with OR ranging between 1.9 and 13.5. However, considerable heterogeneity (I squared statistic >50%) across included studies was documented (Laupacis et al. 2011; Krogias et al. 2013; Tsivgoulis et al. 2014), while a factor contributing to this heterogeneity (according to sensitivity analyses of the largest to date meta-analysis) appears to be the involvement of investigators in endovascular procedures supporting “Liberation treatment” as a novel therapeutic strategy for MS (Tsivgoulis et al. 2014).

### Evaluation of cerebral venous drainage in MS using other than ultrasound neuroimaging modalities

In contrast to the CCSVI hypothesis, three Magnetic Resonance Imaging (MRI) studies failed to document a higher prevalence of impaired cerebral or cervical venous flow due to obstructions in cervical or thoracic veins in MS patients in comparison to controls (Sundström et al. 2010; Wattjes et al. 2011; Blinkenberg et al. 2012). Interestingly, a recent MR venography study failed to find increased prevalence of extracranial venous anomalies in children and adolescents with MS in comparison to healthy controls (Jurkiewicz et al. 2014). Moreover, a contrast venography study in 42 MS patients reported that extracranial venous stenosis is an unlikely cause of MS since it is not present in most patients early in the disease and rarely involves more than one extracranial vein (Yamout et al. 2010).

It should also be noted that ultrasound diagnosis of CCSVI was not confirmed when the same patients were evaluated with another neuroimaging modality including magnetic resonance venography (Blinkenberg et al. 2012; Brod et al. 2013; Costello et al. 2014) or catheter venography (Baracchini et al. 2011; Traboulsee et al. 2014). More specifically, the sensitivity and specificity of the ultrasound criteria for detection of greater than 50% narrowing of any major vein on catheter venography was 40.6% (95% CI: 31.1–50.8%) and 64.3% (48.0–78.0%) in a recent multicenter, double-blinded (blinding of both sonographers and neuro-interventionalists) Canadian study. The highly discrepant results between ultrasound and other neuroimaging modalities lend support to the assumption that CCSVI may constitute a sonographic construct that is unlikely to contribute to MS pathogenesis (Filippi et al. 2011). Finally, Table 3 summarizes the findings of multimodal cross-sectional or case–control neuroimaging studies investigating the CCSVI hypothesis in MS pathogenesis. The majority of these studies failed to validate the CCSVI hypothesis using a combination of different sets of investigations including neurosonology, MR venography, and contrast venography.

**Table 3:** Summary of multimodal neuroimaging studies investigating the CCSVI hypothesis in multiple sclerosis

Study	Imaging modalities	Main findings
Baracchini et al. 2011	DS, CV	• CV did not confirm venous outflow abnormalities in seven CCSVI (+) patients according to DS criteria
Blinkenberg et al. 2012	DS, MRI, PC-MR	• DS and MRI documented no evidence supporting the CCSVI hypothesis
Brod et al. 2013	DS, MRV, TLV	• The three imaging approaches provided generally consistent data not supporting the CCSVI hypothesis
		• No evidence for altered venous outflow in MS patients
Costello et al. 2014	DS, MRV	• DS and MRV documented no evidence supporting the CCSVI hypothesis
Dolic et al. 2011	DS, MRV	• A multimodal noninvasive approach increases the specificity for CCSVI diagnosis in MS patients
Dolic et al. 2012	DS, MRV	• DS is more sensitive than MRV in detecting intraluminal structural and functional venous abnormalities in patients with MS compared with controls
Hojnacki et al. 2010	DS, MRV, CV	• The use of MRV for diagnosis of CCSVI in MS patients has limited value, and the findings should be interpreted with caution and confirmed by other imaging techniques, such as DS and CV
Rodger et al. 2013	DS, MRV	• DS and MRI documented no evidence supporting the CCSVI hypothesis
Simka et al. 2012	DS, CV	• DS criteria for the detection of obstructive venous abnormalities are of limited diagnostic value and diagnosis should be given using CV
Traboulsee et al. 2014	DS, CV	• Although CCSVI occurs rarely in MS patients and controls, extracranial venous narrowing >50% is frequent in both groups
		• The prevalence of CCSVI on CV is low (<5%) in MS patients and healthy controls
		• The DS criteria are neither sensitive nor specific for narrowing on CV
Zivadinov et al. 2011	DS, MRV, CV	• DS showed high specificity and PPV, as well as strong agreement with CV findings at baseline
		• In contrast, conventional MRV had limited value for the detection of venous abnormalities both cross-sectionally and longitudinally
Zivadinov et al. 2012	DS, MRI	• CCSVI is not associated with more severe lesion burden or brain atrophy in MS patients or controls
Zivadinov et al. 2013	DS, MRV, CV	• DS screening was found to be a reliable approach for identifying patients eligible for further multimodal invasive imaging testing

DS, doppler ultrasound; PC-MR, phase-contrast magnetic resonance imaging; MRV, magnetic resonance venography; CV, catheter venography; MRI, magnetic resonance imaging; CP, cervical plethysmography; MS, multiple sclerosis; CCSVI, chronic cerebrospinal venous insufficiency; TVL, transluminal venography; IJV, internal jugular vein; PPV, positive predictive value.

### Methodological shortcomings of proposed ultrasound protocol

The poor reproducibility of CCSVI diagnosis between and within sonographers as well as the low diagnostic yield of ultrasound against other neuroimaging modalities for detection of major cervical vein narrowing may be related to technical reasons including artificial compression of cervical veins by the ultrasound probe or contraction of cervical musculature leading to pseudostenosis, inappropriate selection of pulse repetition frequencies, misinterpretation of pulsation artifact from the adjacent carotid artery as venous reflux, failure to recognize intraluminal jugular septation causing IJV stenosis, misinterpretation of IJV valve insufficiency as IJV stenosis, inadequate patient compliance during sonographic evaluation of cervical veins at different body positions and during different phases of breathing (Baracchini et al. 2011; Tsivgoulis et al. 2011; Valdueza et al. 2013). Another plausible explanation may be associated with the incomplete blinding of the investigators and potential variabilities in the hydration status of MS patients (Comi et al. 2013). In addition, it is also clear that there are enormous variations in normal patterns of cerebral venous drainage within the healthy population, and that interpretation of patterns of venous drainage and venous obstruction can be highly subjective, subject to observer bias, and discrepant from institution to institution, depending upon the particular technology used for assessment as well as the expertise of the sonographers in cerebral and cervical venous ultrasound examinations (Valdueza et al. 2013). The potential methodological shortcomings of the proposed neurosonology protocol of CCSVI detection are summarized in Table 4. Finally, Table 5 displays a critical appraisal of available neurosonology images in the pivotal publications by Zamboni and colleagues introducing the CCSVI hypothesis.

**Table 4:** Methodological shortcomings of proposed neurosonology protocol for CCSVI diagnosis (Baracchini et al. 2011; Tsivgoulis et al. 2011; Valdueza et al. 2013)

Criterion	Methodological shortcoming
(I): Reflux in cervical veins	1 The threshold of 0.88 sec for diagnosing “reflux” in cervical veins has been validated only for internal jugular valve (IJV) valve insufficiency [no validation for Vertebral Veins (VV)]
	2 Nonpathologic oscillating signal with positive and negative flows can be observed in IJV especially in the oldest old [pulsation of internal carotid artery (ICA)] leading to false-positive diagnosis of extracranial venous reflux
	3 Two different time values have been used to define extracranial (0.88 sec) and intracranial (0.55 sec) reflux
(II): Reflux in deep cerebral veins	1 Introduction of a novel acoustic window by Zamboni termed “supracondylar” (substituting classic transtemporal window)
	2 Evaluation of intracranial venous reflux using only Color-Coded Mode analysis (nonmandatory Doppler spectrum analysis) leading to false-positive diagnosis of intracranial venous reflux
	3 The threshold of reflux (0.55sec) was arbitrary and was derived from studies evaluating venous insufficiency in the legs
	4 The detection rate of internal cerebral vein (10–20%), sigmoid sinus (20–50%), Vein of Galen (30–60%) using Transcranial sonography is low and consequently these cerebral veins and sinuses cannot be evaluated with ultrasound in a substantial portion of patients
(III): High-resolution B-mode evidence of proximal IJV stenosis	1 The cutoff value of IJV stenosis (cross-sectional area<0.3 cm^2^) was derived from a study evaluating patients in Intensive Care Unit (never studied in healthy controls)
	2 Physiologic dilatations of IJV (superior & inferior bulb) may lead to false-positive diagnosis of IJV stenosis distal to the dilatation
	3 No definition of location of the designated normal reference
	4 Cervical vein compression by probe or contraction of sternocleidomastoid muscle and intraluminal septation of IJV valve may lead to false-positive diagnosis of IJV stenosis
	5 Cervical venous drainage is dominated by right side and hypoplastic left IJV is a common anatomic variation that may be misdiagnosed as IJV stenosis
(IV):Flow not-Doppler detectable in IJV and or VVs	1 Absent flow in IJV (upright position) or in the Vertebral veins (supine position) does not reflect pathologic condition and has been described in healthy controls
	2 Cervical vein compression by probe or contraction of sternocleidomastoid muscle, incorrect (high) pulse repetition frequency settings may lead to false-positive diagnosis of flow not-Doppler detectable in IJV
(V):Reverted postural control of the main cerebral venous outflow in IJVs	1 Technical challenging (mild compression by probe or muscle contractions may affect IJV diameter leading to false-positive results)
	2 IJV may be completely collapsed in upright position. Deep neck veins and subclavian vein may be misidentified as IJV
	3 Cross-sectional area of IJV may be affected by breathing, neck position, and slight patient movements during insonation leading to low reproducibility of cross-sectional area measurements

**Table 5:** Critique of published neurosonology images in pivotal studies introducing CCSVI hypothesis (Zamboni 2006; Zamboni et al. 2009a,b,c)

Criterion	Methodological Shortcoming
(I): Reflux in cervical veins	Doppler interrogation was not performed simultaneously in Color-Coded images demonstrating venous reflux in cervical veins. Only color-coded images in the transverse section (without complementary color-coded images in horizontal section) were provided. Alternatively, only color-coded images in the horizontal section (without complementary color-coded images in transverse section) were provided
(II): Reflux in deep cerebral veins	Doppler interrogation was not performed simultaneously in Color-Coded images demonstrating venous reflux in cervical veins. Anatomical landmarks of brain parenchyma that may assist in the correct identification of intracranial vessels were not depicted. Deep middle cerebral vein is presented as a vein located in subcortical gray matter without any further identifying details
(III): High-resolution B-mode evidence of proximal IJV stenosis	No images of the location of the designated normal reference were provided. There is no comment with regard to the exclusion of left IJV (internal jugular vein) hypoplasia, a common anatomic variation in extracranial veins that may have resulted in false-positive findings in the displayed images
(IV): Flow not-Doppler detectable in IJV and/or VVs	No images with such pathology in vertebral veins (VVs) were provided. Only color-coded images of IJVs in the transverse section (without complementary color-coded images in horizontal section) were provided. Alternatively, only color-coded images of IJVs in the horizontal section (without complementary color-coded images in transverse section) were provided
(V): Reverted postural control of the main cerebral venous outflow in IJVs	No images of quantitive studies of blood flow volumes measurements were provided in order to substantiate the hypothesis reverted postural control. No images of the different patient positions during ultrasound measurements were provided

### Safety and efficacy of “Liberation treatment”

An argument postulated by several groups including patient advocacy groups, media representatives, and physicians is that even with suboptimal accuracy parameters a positive therapeutic effect of venous angioplasty and/or stenting cannot be excluded in MS patients with CCSVI constellations (Pullman et al. 2013; Zivadinov et al. 2013). Consequently, they postulate that access to such interventional therapies should not be refused in the context of randomized-controlled trials, while additional funding and intellectual energy are required to further investigate the venous hypothesis of MS pathogenesis (Zivadinov et al. 2013).

In contrast, there is growing literature underscoring that “Liberation treatment” for CCSVI can be complicated by serious adverse events including IJV or cerebral venous thrombosis, stent dislocation, vein dissection, femoral artery pseudoaneurysm, cranial nerve palsies, syncope or severe cardiac arrhythmias, hydrocephalus, and hemorrhagic complications of anticoagulation initiated following stent placement (Burton et al. 2011; Ghezzi et al. 2013a; Tsivgoulis et al. 2014). Table 6 summarizes the reported complications of “Liberation treatment” across different studies.

**Table 6:** Reported complications of “Liberation treatment” for treatment of CCSVI in multiple sclerosis patients

Study	Description of complication
Zamboni et al. (2009c)	No major complication reported. Mild postprocedural headache with spontaneous resolution (*n* = 6) Minor hemorrhages (hematomas) at the vascular access sites (exact number not reported)
Samson (2010)	Fatal brainstem hemorrhage in a patient treated with coumadin following insertion of two self-regulating stents in the right internal jugular vein (IJV, *n* = 1). Migration of stent placed in IJV to the right ventricle. Open heart surgery was performed to remove the device (*n* = 1)
Ludyga et al. (2010)	Stent thrombosis (*n* = 2). Surgical removal of angiographic balloon (*n* = 1). Local bleeding from groin (*n* = 4). Two cases with femoral artery pseudoaneurysm treated with thrombin injection (*n* = 2). Gastro-intestinal bleeding requiring hospitalization following clopidogrel treatment after stent placement (*n* = 1). Transient atrial fibrillation during the procedure requiring pharmacological treatment (*n* = 2). Migration of stent placed in IJV (*n* = 4). Second stent placement required to secure the first one (*n* = 4)
Thapar et al. (2011)	IJV thrombosis following venoplasty (*n* = 1). Open thrombectomy performed for symptom relief
Burton et al. (2011)	IJV thrombosis following stent placement (*n* = 1). Cranial nerve palsies (hypoglossal and accessory nerves) caused by bilateral oversized stent placement in IJV (*n* = 1) Migration of stent from azygos to renal vein causing syncope (*n* = 1) Surgical dissection of femoral vein during balloon withdrawal causing large extraperitoneal hematoma within the space of Retzius leading bladder compression (*n* = 1) IJV thrombosis following stent placement complicated by thrombosis of ipsilateral transverse and sigmoid sinuses (*n* = 1) Anticoagulation was required to treat iatrogenic cerebral venous sinus thrombosis
Petrov et al. (2011)	Limited groin hematoma (*n* = 5, cardiac arrhythmias (*n* = 6), vein rupture (*n* = 2), vein dissection (*n* = 15), acute in-stent/in-segment thrombosis (*n* = 8), and acute recoil (*n* = 1)
Hubbard et al. (2012)	Deep vein thrombosis at the venous access site (*n* = 1)
Doležal et al. (2012)	Dislocation of right IJV stent to ipsilateral brachiocephalic vein and thrombosis of left IJV stent requiring anticoagulation (*n* = 1)
Zamboni et al. (2012)	Vasovagal syncope reported 3 h after procedure (*n* = 1)
Mandato et al. (2012)	Neck pain (*n* = 40), venous thrombosis requiring retreatment within 30 days (*n* = 3), sustained intraprocedural arrhythmias requiring hospitalization (*n* = 2), stress-induced cardiomyopathy requiring hospitalization (*n* = 1)
Ghezzi et al. (2013a)	IJV thrombosis (*n* = 7), tetraventricular hydrocephalus that needed temporary shunting (*n* = 1), stroke (*n* = 1), paroxysmal atrial fibrillation (*n* = 1), status epilepticus (*n* = 1), aspiration pneumonia leading to subsequent postanoxic encephalopathy (*n* = 1), hypertension with tachycardia (*n* = 1), severe bleeding of bedsore due to anticoagulation treatment following the procedure (*n* = 1)
Barbato et al. (2014)	Bilateral IJV thrombosis leading to occlusion of right IJV and severe stenosis of left IJV in a patient who underwent four procedures of bilateral IJV angioplasty and stenting for restenosis
Siddiqui et al. (2014)	Cardiac event (24 h after procedure) treated with pacemaker installation (*n* = 1) Swelling and soarness at the side of the neck of venous angioplasty (*n* = 1)

Finally, a recently published class I sham-controlled, randomized, double-blind study investigating the safety and efficacy of venous angioplasty in MS patients fulfilling ultrasound criteria of CCSVI showed that venous angioplasty did not improve hemodynamic parameters in terms of venous hemodynamic insufficiency severity score as well as clinical outcomes in terms of annualized relapse rate, Expanded Disability Severity Score, and MS Functional Composite (Siddiqui et al. 2014). Moreover, “Liberation treatment” exacerbated underlying disease activity in terms of new T2 and gadolinium-enhancing T1 lesions (Siddiqui et al. 2014). In line with these findings, two recent open-label, retrospective studies also showed an increase in disease activity irrespective of adherence to disease-modifying therapies in patients with MS with CCSVI who underwent venous angioplasty (Alroughani et al. 2013; Ghezzi et al. 2013a).

## Conclusions

The postulated hypothesis of a disturbed mechanical cervicospinal venous drain as a monofactorial etiopathogenic mechanism of MS should be discarded in view of the numerous independent external validation studies contradicting Zamboni's observations and the highly discrepant findings between ultrasound and other neuroimaging modalities. CCSVI appears to be a poorly reproducible and clinically irrelevant sonographic construct. “Liberation treatment” has no proven efficacy, may exacerbate underlying disease activity and has been complicated with serious adverse events. “Liberation treatment” should stop being offered to MS patients even in the settings of randomized-controlled trials, while further unnecessary expenditure of scarce funding resources needs to be discontinued. Physicians taking care of individuals with MS should spend time educating their patients with regard to the scientific evidence refuting CCSVI hypothesis as well as the potential complications and the lack of efficacy of “Liberation” treatment replacing the blogosphere as the main source of “sensational” but inaccurate information.
